# Industrial Hygiene

**Published:** 1920-05-15

**Authors:** E. H. Snell

**Affiliations:** Recording Secretary, Industrial Hygiene Section, Birmingham Congress of the Royal Sanitary Institute.


*To the Editor of* The Hospital.

### Industrial Hygiene.

*To the Editor of* The Hospital.

Sir,—The letter in your issue of May 1, appearing above the name of Miss Gladys M. E. Leigh, prompts me to write to the effect that in the Industrial Hygiene Section of the Birmingham Congress next July the Institute is arranging for a discussion of welfare work in factories. If any of your readers, nurses or others, desire to read a paper or join in a discussion on this or kindred matters, I shall be glad if they will communicate with me, and I will make arrangements accordingly.—Yours faithfully,

E. H. Snell,
Recording Secretary, Industrial Hygiene Section,
Birmingham Congress of the Royal Sanitary Institute.

